# MetaBinG2: a fast and accurate metagenomic sequence classification system for samples with many unknown organisms

**DOI:** 10.1186/s13062-018-0220-y

**Published:** 2018-08-22

**Authors:** Yuyang Qiao, Ben Jia, Zhiqiang Hu, Chen Sun, Yijin Xiang, Chaochun Wei

**Affiliations:** 1 Department of Bioinformatics and Biostatistics, School of Life Sciences and Biotechnology, Shanghai Jiao Tong University, Shanghai, 200240 China; 2 Shanghai Center for Bioinformation Technology, Shanghai, 201203 China; 3 Shanghai Center for Systems Biomedicine, Shanghai Jiao Tong University, Shanghai, 200240 China; 4 School of Medicine, Shanghai Jiao Tong University, Shanghai, 200025 China

**Keywords:** Metagenome, MetaSUB, Sequence classification

## Abstract

**Background:**

Many methods have been developed for metagenomic sequence classification, and most of them depend heavily on genome sequences of the known organisms. A large portion of sequencing sequences may be classified as unknown, which greatly impairs our understanding of the whole sample.

**Result:**

Here we present MetaBinG2, a fast method for metagenomic sequence classification, especially for samples with a large number of unknown organisms. MetaBinG2 is based on sequence composition, and uses GPUs to accelerate its speed. A million 100 bp Illumina sequences can be classified in about 1 min on a computer with one GPU card. We evaluated MetaBinG2 by comparing it to multiple popular existing methods. We then applied MetaBinG2 to the dataset of MetaSUB Inter-City Challenge provided by CAMDA data analysis contest and compared community composition structures for environmental samples from different public places across cities.

**Conclusion:**

Compared to existing methods, MetaBinG2 is fast and accurate, especially for those samples with significant proportions of unknown organisms.

**Reviewers:**

This article was reviewed by Drs. Eran Elhaik, Nicolas Rascovan, and Serghei Mangul.

**Electronic supplementary material:**

The online version of this article (10.1186/s13062-018-0220-y) contains supplementary material, which is available to authorized users.

## Background

With the greatest biodiversity and huge quantity, microbes occupy a very important position in the ecosystem. However, most of them have not been studied through traditional separate-and-culture methods [1], since only a small fraction of them are culturable. Metagenomics provides a culture-independent method to study an environment by sequencing the genetic material directly. With the progress of sequencing technologies, some environments such as gut microbiomes have been studied well. However, in most environments, most microbes are unknown and were ignored in the current studies [2]. Metagenomics analysis of unknown environments may give us brand new view points and tremendous genetic resources. For example, health and disease can be determined by the diversity patterns of the human microbiomes [3]. The microbial diversity in marine can provide an accurate index of environmental health and ecosystem sustainability [4]. The study of microbial communities with high diversities in soil is helpful to understand the important process related with the plant growth and cycling of carbon [5]. Metagenome sequence analysis can help for all these diverse research areas.

Sequence classification is a crucial step in metagenome sequence analysis. The methods for metagenome sequence classification can be divided into two categories: (1) alignment-based methods and (2) composition-based methods. Alignment-based methods can be further divided into seed-and-extend alignment-based method, mapping-based methods and kmer-alignment based methods. Seed-and-extend alignment-based methods like BLAST [6] and DIAMOND [7], which classify a query sequence by finding the best alignment to a big database of reference genomes through sequence alignment methods. DIAMOND uses double indexing which determines the list of all seeds and their locations in both the query and reference database. Mapping-based methods are faster than seed-and-extend alignment-based methods because of the benefits from the mapping algorithm, while their sensitivity is lower in general, like MiCoP [8]. Kmer-alignment-based methods, like KRAKEN [9] and CLARK [10], have advantages both on speed and precision by using exact-match of kmers, rather than inexact alignment of sequences, to the reference database. For example, KRAKEN is about 900 times faster than Megablast (BLAST-based system) [9]. However, for all these alignment-based methods, their accuracy drops dramatically when dealing with samples with many unknown organisms. By contrast, composition-based methods, such as Phymm [11], NBC [12] and metaCV [13] depend less on reference genomes. Phymm uses interpolated Markov models (IMM) to characterize variable-length oligonucleotides for phylogenetic grouping. NBC uses the Naive Bayes method to classify sequences to their best taxonomic group. MetaCV uses k-mer frequency vectors of translated peptide sequences instead of the nucleotide sequences against the reference protein sequence database to determine the source organism. In summary, compared with alignment-based methods, composition-based methods have low dependence on the reference genomes, but at the same time, their accuracy is lower in general.

GPUs (Graphic processing units) were originally designed to accelerate graphic display but can be utilized for some scientific computing. GPUs have advantages on numerical calculation benefited from the hundreds of cores. With the success of CUDA, a parallel programming model designed for GPU [14], many applications, including some in bioinformatics, have obtained considerable acceleration by adapting GPUs [15]. In this paper, we present a composition-based method - MetaBinG2, together with its GPU version, for metagenome sequence classification and a toolkit named MetaBinG2kit to visualize the analysis results. The performance of MetaBinG2 were tested on simulated and mock datasets. In the end, MetaBinG2 was applied to the dataset of MetaSUB Inter-City Challenge provided by CAMDA data analysis contest [16] and the community composition structures for environmental samples from different public places across three cities have been analyzed and compared.

## Methods

Two reference datasets and four query datasets were prepared to evaluate the performance. The two reference datasets were denoted as reference dataset A and B. Reference dataset A and multiple reference databases derived from it were designed for performance evaluation. Dataset B was prepared for real-world data analysis for large-scale metagenome sequencing projects, like MetaSUB.

The four query datasets were: i.) Simulated dataset, ii.) Mock dataset, iii.) Cow Rumen dataset, and iv.) MetaSUB dataset. The first two datasets were used to evaluate the methods in terms of classification accuracy, and the running speed. Cow Rumen dataset was used to show the results of several methods when they were applied to classify real-world samples with many unknown organisms. MetaSUB dataset was used to test MetaBinG2’s application ability for large-scale metagenome sequencing projects.

### Reference dataset A

Reference dataset A contains 2606 microbe genomes and the genome numbers at various taxonomy level are shown in Table 1. They were downloaded from NCBI website (ftp://ftp.ncbi.nlm.nih.gov/genomes/archive/old_refseq/Bacteria/, updated on June 2, 2015). Multiple databases were generated from this reference dataset A to evaluate CLARK, DIAMOND, metaCV, MetaBinG, and MetaBinG2. All reference databases in our analysis except for MetaSUB analysis were generated according to Reference dataset A.

**Table 1:** The details about genomes included in the reference datasets

	Phylum	Class	Order	Family	Genus	Species	Genome
Reference dataset A	38	63	147	265	690	1429	2606
Reference dataset B	43	87	188	357	958	2733	7675

### Reference dataset B

Reference dataset B is a comprehensive reference dataset. It contains 7675 genomes, including 7459 from bacteria, 63 from eukaryotes, 153 from Archaea. These genomes were downloaded from NCBI genome database (ftp://ftp.ncbi.nlm.nih.gov/genomes/, updated on Mar 27, 2017). The bacterial genome numbers at various taxonomy levels are shown in Table 1. Reference dataset A is a subset of reference dataset B. A comprehensive database was generated from this reference dataset B for MetaBinG2 on the MetaSUB dataset.

### Simulated datasets

Simulated metagenome sequencing datasets were created as inputs. The community composition structure information of the simulated metagenome sequencing data comes from a published work [17]. We used NeSSM [18] to simulate 100 million single sequences with sequence length of 100 bp and 250 bp according to the community composition structure (Additional file 1: Figure S1).

### Mock dataset

Another way to evaluate metagenomics analysis methods is using a mock dataset, which is generated by sequencing a mock community (a mixture of microbes with predefined proportions). In terms of similarity to the real-world data, a mock data is between simulation data and real-world metagenome sequencing data. We downloaded a mock dataset from HMP Microbiome Mock Community (HMMC, SRA run id: SRR072232). In this mock dataset, not all species are with the same proportion. Some species are dominant in this mock dataset (see details in Additional file 1: Table S1).

### Cow rumen dataset

We chose a real-world dataset which was generated from the cow rumen [19] (SRA runid: SRX034809). The sample was sequenced by Illumina GAIIx with sequence length of 125 bps. The total number of sequences is about 140 million.

### MetaSUB dataset

The MetaSUB dataset is also known as CAMDA 2017 conference - challenge two. This dataset was generated from metagenomes sampled from subway stations of three cities: Boston, New York (NY), and Sacramento. Different locations of the subway stations were sampled. MetaSUB data is a real-world large-scale metagenome sequencing data. The size of the sequencing data in fastq format is about 3.6 TB. Considering the high complexity of this dataset, to better analyze the data, we used a much more comprehensive reference database B, including a bigger number of prokaryotic genomes and some additional eukaryotic genomes (see beginning of the Methods section for more details).

### Method evaluation

We evaluated MetaBinG2 in three aspects: (1) classification accuracy, (2) community composition structure prediction ability and (3) running speed. This was done by comparing MetaBinG2 to several existing methods: alignment-based method – CLARK and DIAMOND, composition-based method – metaCV, and the first version of MetaBinG2 – MetaBinG.

1. Classification accuracy

**Fig. 1:**
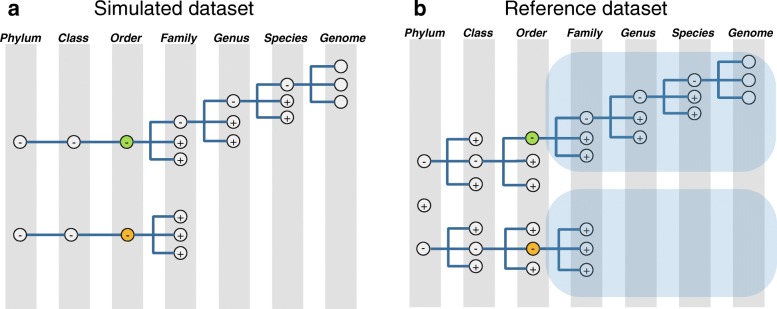
Schematic diagram of clade exclusion experiment. **a** is a diagram of the community composition structure in a simulated query dataset. All genomes in the simulated query dataset are from the two orders represented by the nodes colored with ‘green’ and ‘yellow’. **b** is a diagram of creating ‘Order_excluded’ reference database. All nodes in (**b**) stands for the original reference dataset A. Nodes colored with ‘green’ and ‘yellow’ are corresponding to the ones in (**a**) with same colors. The genomes under the nodes which are covered by the light blue part are excluded from reference dataset A to construct ‘Order_excluded’ database. In this figure, ‘+’ means that the inferior details are condensed, and ‘-’ means these details are expanded

We used clade exclusion experiments with simulated dataset and reference dataset A to evaluate the classification accuracy. Clade exclusion experiments were used to evaluate methods’ ability to classify the samples with different degree of unknown organisms. We generated several reference databases with different clade exclusion by modifying the reference dataset A according to the known community composition structure of simulated query dataset to mimic metagenome analysis with unknown organisms. For example, to create a scenario with unknown organisms at order taxonomy level, we generated ‘Order_excluded’ reference database by excluding from the reference dataset A those genomes with the same order as those in the query dataset. More details of this process are illustrated in Fig. 1. As a result, we got six reference databases for simulated query dataset: (1) ‘No_exclusion’ reference database which is the same as the original reference database A (with 2606 genomes); (2) ‘Species_excluded’ database (with 2557 genomes); (3) ‘Genus_excluded’ database (with 2436 genomes), (4) ‘Family_excluded’ database (with 2153 genomes), (5) ‘Order_excluded’ database (with 1965 genomes), and (6) ‘Class_excluded’ database (with 550 genomes). Databases (2) - (6) stand for different degrees of unknown organisms in a sample.

We used several accuracy measurements for the method evaluation. ‘TP’ represents the number of sequences that their predicted taxonomies were the same as their true taxonomies. ‘UN’ represents the number of unclassified sequences. ‘ALL’ represents the total number of sequences. We calculated sensitivity = TP/ALL, precision = TP/(ALL-UN), and accuracy = (sensitivity + precision)/2.

(2) Community composition structure prediction ability

We used simulated dataset and mock dataset with reference dataset A to compare community composition structure prediction accuracy for several metagenome sequence classification tools. The consistency between a predicted community composition structure and the expected community composition structure was measured by cosine distances at different taxonomy levels.

We also calculated the over-prediction rates at different taxonomy levels. The community composition structures were known for simulated datasets and mock datasets. The over-prediction rate was computed as the percentage of predicted taxonomy items not included in the expected taxonomy items, i.e. the number of predicted taxonomy items not included in the expected composition structure divided by the total number of predicted taxonomy items.

We calculated Shannon index to reflect the community diversity of each sample in the analysis of MetaSUB dataset. The formula for Shannon index is described as follows.

1 $$ H=-\sum \limits_{i=0}^N{p}_i\mathit{\ln}{p}_i $$

(3) Running speed and memory requirement

Since the tools used for comparison are fast, we tested all of them in one machine to compare their speed.

### Method of MetaBinG2

1. Building reference database

For genomes in the reference dataset, MetaBinG2 converts a complete genome sequence into a state-transition probability vector of the *k*^th^-order Markov model. A state in this Markov model is defined as a sequence of length *k*, and each state can transfer to four states, so that there are 4^(*k* + 1)^ transition probabilities. The transition probabilities from a state *m* to a state *n* of the genome *i* is calculated as following:

2 $$ {KMM}_{i, mn}={P}_i\left(\operatorname{}{O}_n|{O}_m\right)=\frac{F_i\left(\operatorname{}{O}_n|{O}_m\right)}{F_i\left({O}_m\right)} $$

Where *O*_*m*_and *O*_*n*_are oligonucleotides of length *k* with *k* − 1 bases overlapped, *F*_*i*_(*O*_*m*_) stands for the number of state *m* in genome *i*, *F*_*i*_(*O*_*n*_| *O*_*m*_) stands for the number of state *m* followed by state *n* in genome *i*, and *P*_*i*_(*O*_*n*_| *O*_*m*_) represents the transition probability from the *O*_*m*_ to the *O*_*n*_ of the genome *i*.

(2) Calculating the similarity scores between a short sequence and the reference genomes

We designed MetaBinG2 based on an assumption that a query sequence is more likely from the organism with a larger proportion when the similarity scores of a query sequence to several organisms are similar. The similarity score between a short sequence with length *l* and a genome *i* can be measured by a score *S*_*i*_ as following:

3 $$ {S}_i=\left(-\sum \limits_{j=0}^{l-k-1}\mathit{\ln}\left({p}_i\left(\left.{O}_{j+1}\right|{O}_j\right)\right)\right)\ast \left(1+{\upomega}_i\right) $$

where *O*_*j*_ and *O*_*j* + 1_ are oligonucleotides of length *k*; *p*_*i*_(*O*_*j* + 1_| *O*_*j*_) represents the transition probability from the *O*_*j*_ to the *O*_*j* + 1_ of the genome *i*; ω_*i*_ stands for the weight of genome *i* which is calculated according to the number of sequences assigned to genome *i*. Here, *k* is set to be 5 because MetaBinG got a good performance with balanced accuracy and speed when *k* is 5 [20]. The similarity scores between the query sequences and reference genomes were computed through a matrix multiplication, which is achieved by using CUBLAS library [21] on GPU. The improvement of algorithm is reflected with the value of ω_*i*_. Each genome will be assigned with an initial value (an evenly distributed probability) at the beginning. These values will be updated when a classification is completed and as the prior knowledge added in the next classification. We obtained the final classification result when this genome weights vector ω is leveled off. At the end, the genome with the best similarity score will be assigned to each query sequence, and its taxonomy information will be output.

**Fig. 2:**
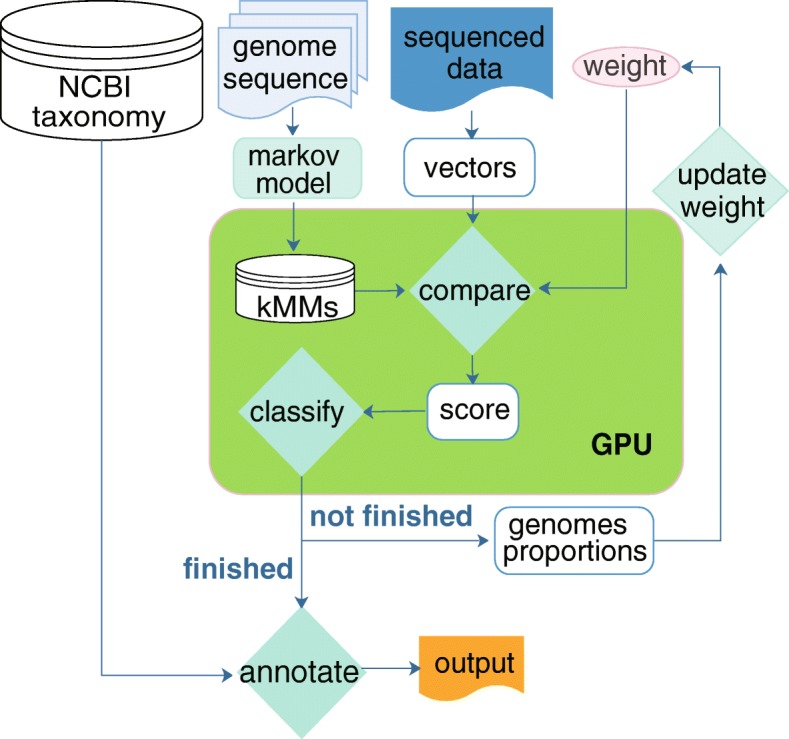
The system diagram of MetaBinG2. MetaBinG2 first loads the reference database and copy it into GPUs as a reference matrix. Next, the short query sequences are converted into k-mer vectors in CPUs, and vectors will be loaded to GPUs as query matrix. Then, the query matrix will be multiplied to the reference matrix in GPUs by CUDA CUBLAS functions and adjusted with the weights, with a similarity score matrix as the output. The source genomes with minimum similarity scores will be selected. The weights are updated according to the latest proportions after all sequences are classified. If the BC distances of the weights before and after the update are less than the cutoff, the final similarity scores together with the annotated taxonomy information will be output

MetaBinG2 with GPU classification system is shown in Fig. 2. The similarity scores between the query sequences and reference genomes were computed in GPUs. After the scores were computed, the source genome with minimum score was assigned to a query sequence in CPUs. In practice, query sequences are loaded into GPUs in batches. By default, each batch have 1000 query sequences.

### Development environment

MetaBinG2 was developed with CUBLAS library (CUDA 7.5) and pthread library on a Linux machine with 16 CPU cores (Intel (R) Xeon (R) CPU E5-2680 v3 @ 2.50GHz) and two Tesla K80 GPU cards (only one GPU was used for all MetaBinG and MetaBinG2). All other tools were tested on the same machine.

## Results

We have implemented MetaBinG2 program for metagenomic sequence classification. Its performance was evaluated on simulated sequencing datasets and a mock dataset. The scenarios for samples with unknown organisms were simulated by clade exclusion experiments (Fig. 1). MetaBinG2 was then applied to analyze two real-world datasets: Cow Rumen dataset and MetaSUB dataset.

### Clade exclusion experiments

**Fig. 3:**
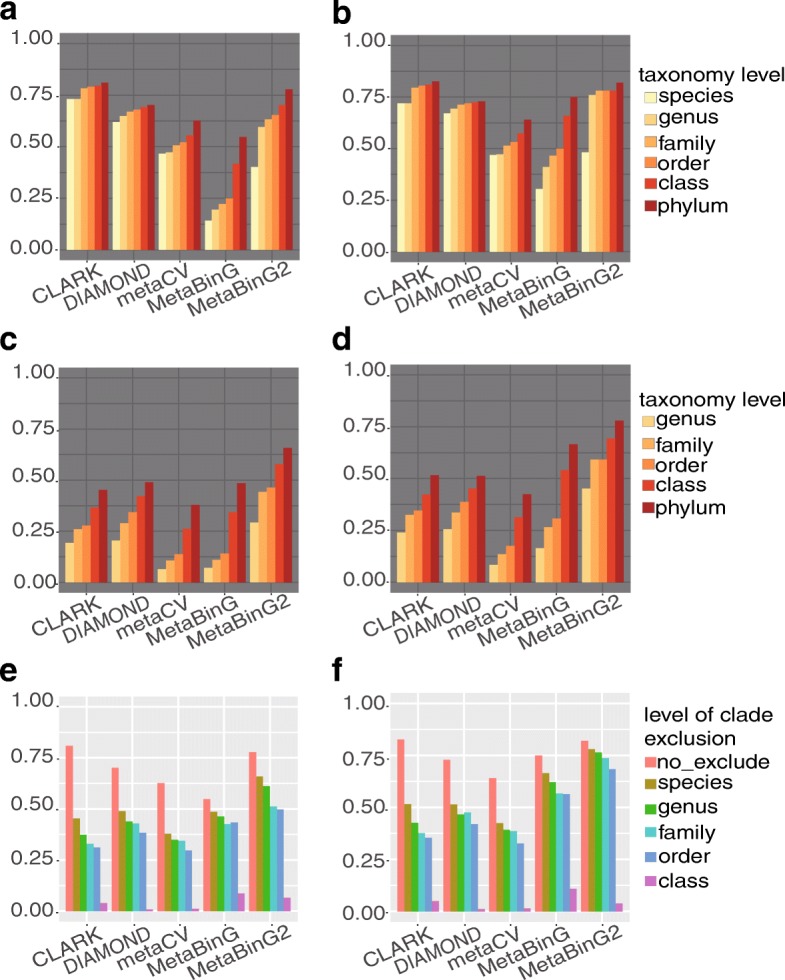
Accuracy evaluation with simulated datasets against reference databases with/without clade exclusion. Tested scenarios include: **a**, **b** no exclusion, **c**, **d** species level clade exclusion, and **e**, **f** all level of clade exclusion. In **a**, **b**, all genomes in the query datasets were included in the reference database and query sequence length is 100 bp (**a**) and 250 bp (**b**); In **c**, **d** all species in the query datasets were excluded in the reference database. The accuracy was measured on clade levels from species to phylum (**a**-**d**). **e**, **f** accuracy evaluation at phylum level, and different levels of clades were excluded in the reference database. In **a**, **c**, **e** the query sequence length is 100 bp, and in **b**, **d**, **f** the query sequence length is 250 bp. Y-axis in all Fig. 3 stands for the accuracy (see details in Methods). Here, CLARK and DIAMOND are alignment-based methods and the others are composition-based methods

For ‘No_exclusion’ experiments, all genomes in a sample have at least one closely related genome in the reference database. CLARK had the best accuracy on all taxonomy levels (Fig. 3a). MetaBinG2 had similar accuracy as CLARK and DIAMOND on phylum level, and showed notable improvement compared to MetaBinG. While at species level, MetaBinG2 was not as good as CLARK and DIAMOND (Fig. 3a). However, when there were unknown genomes, MetaBinG2 performed much better than all other methods (Fig. 3c-d). In addition, the performance of MetaBinG2 was more robust than existing methods for samples with various degrees of unknown genomes and was better as the length of sequencing sequences increases. For example, the evaluation at phylum level was shown in Fig. 3e-f, and results at the other taxonomy level were shown in Additional file 1: Figure S2.

### Consistency between the predicted community composition structure and the expected ones

**Fig. 4:**
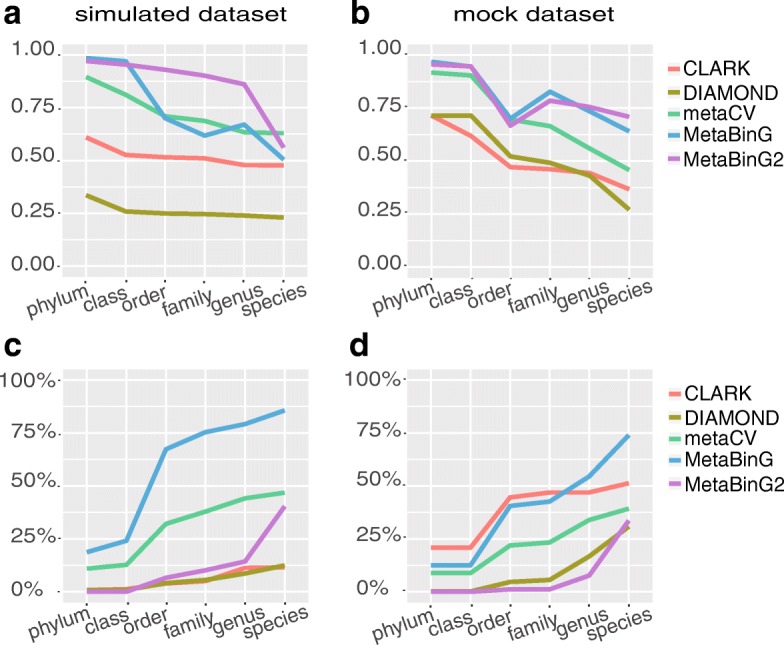
Evaluation for community composition structure prediction ability of each tool. **a**, **b** The consistency between the predicted community composition structure and the true community composition structure. Y-axis stands for consistency level reflected by cosine value. In **a** the query dataset was simulated dataset (with sequence length of 100 bp), and in **b** the query dataset was mock dataset. **c**, **d** Over-prediction of each tools. The Y-axis stands for the proportion of query sequences assigned to genomes outside of the true source genomes (not include the unclassified part). In **c** the dataset was simulated dataset (with sequence length of 100 bp), and in **d** the dataset was mock dataset with known composition structure. Here, CLARK and DIAMOND are alignment-based methods and the others are composition-based methods

We evaluated the consistency between community composition structure predicted by the selected tools and the true composition structures of simulated dataset or mock dataset. The performance of MetaBinG2 is the best based on the consistency between the predicted community composition structure and the expected ones (Fig. 4a-b). For the mock dataset, its gold standard community composition structure was estimated according to its gDNA content (Additional file 1: Table S1). Similar analysis has been done on simulated dataset (with sequence length of 100 bp) with ‘Species_excluded’ reference database and ‘Genus_excluded’ database (Additional file 1: Figure S3). The over-prediction rates of these tools with simulated dataset and mock dataset were shown in Fig. 4c-d. The source genome of each sequence in the mock dataset was unknown, but the mock dataset had known composition structure so that we could evaluate the tools on this dataset through over-prediction rates. The over-prediction rate is the ratio of predicted taxonomy items not included in the expected composition structure and all predicted results without ‘unclassified part’. This rate reflected how many taxa predicted were not included in the list of taxa used for test dataset generation. MetaBinG was prone to predict more wrong taxa results with over 50% on genus level. The performance of MetaBinG2 was much better than MetaBinG and similar to DIAMOND.

### Speed and memory requirement

We applied these selected tools to a simulated dataset with 100 million sequences of length 100 bp against the reference database A (2,606 genomes) on a same machine (see details in methods). The time was measured in two parts, the time for loading database and the time for classifying. Results showed that CLARK was the fastest, while MetaBinG2 was comparable with DIAMOND and slightly better than metaCV and MetaBinG in terms of speed (Table 2). In addition, the memory required for CLARK and metaCV was more than 50GB, for DIAMOND was 23GB but for MetaBinG and MetaBinG2 was less than 1GB. Therefore, MetaBinG2 has a great potential to include many more genomes in the reference database than the other programs.

**Table 2:** The speed and memory requirement

		CLARK	DIAMOND	metaCV	MetaBinG	MetaBinG2
Time (second)	Loading	85	301	722	6	6
	Classifying	375	5240	10,429	7223	5052
Memory (Byte)		>50G	23G	>50G	230 M	510 M
CPU usage		1600	1556	1600	106.2	356.2
CPU time (second)		42	1275	2818	117	261

The four tools were tested on the simulated dataset with 100 million sequences with length 100 bp against the reference database A

### Applying MetaBinG2 to cow rumen dataset

The dataset of cow rumen was a real-world environmental metagenome and contained a large proportion of unknown organisms. Previous researchers produced 15 near-complete draft genomes by an assembly method and assigned them into four orders, Bacteroidales, Clostridiales, Myxococcales, and Spiochaetales [19]. The corresponding classes are Bacteroidia, Clostridia, Deltaproteobacteria, and Spirochaetia and the phyla are Bacteroidetes, Firmicutes, Proteobacteria, and Spirochaetes.

We ran CLARK, DIAMOND, metaCV and MetaBinG2 on this cow rumen dataset with reference dataset A as the reference database. The four orders were all included in MetaBinG2’s prediction results (Additional file 1: Figure S4). However, alignment-based methods, like CLARK and DIAMOND, had a large part of unclassified results labeled as ‘NA’ when they were applied on a sample which has many unknown organisms such as cow rumen dataset. CLARK could not classify ~ 60% sequences of this dataset and DIAMOND could not classify ~ 90% (Additional file 1: Figure S4). This showed the performance of each tool when they were applied on the sample with many unknown organisms. MetaBinG2 is helpful to learn the community composition structure roughly in a short time when we have little knowledge about an environment.

### Applying MetaBinG2 to MetaSUB dataset

We used MetaBinG2 to classify the whole MetaSUB dataset with reference dataset B including eukaryotic genomes described before and we were able to finish the analysis within 3 days using 38 computational nodes (in a high performance computer cluster). The classification results of MetaSUB dataset were listed in Additional files 2, 3 and 4.

MetaSUB includes metagenomic samples from three cities. Relevant information about these samples including the number of samples for each city, average number of sequences per city and standard deviation was described in Additional file 1: Table S2.

**Fig. 5:**
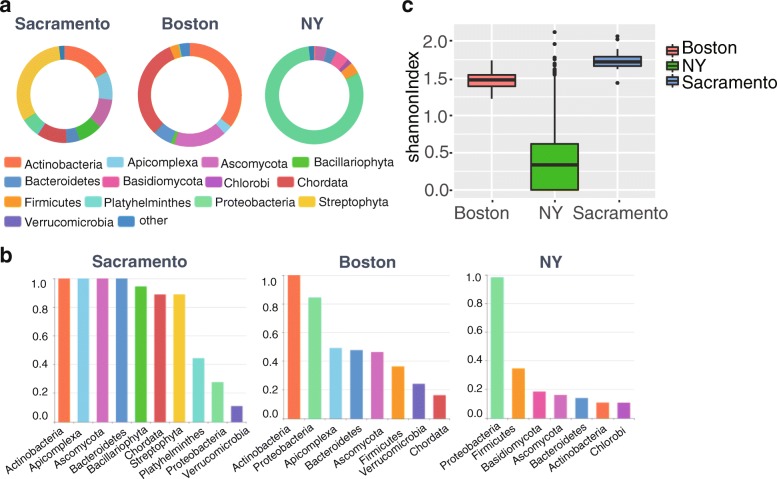
Comparison of community composition structures derived from metagenome samples from different cities. **a** Average community abundance of three cities at phylum level. The number of samples for each city is 18 (Sacramento), 24 (Boston) and 1451 (NY). **b** The proportion of samples containing a certain phylum. Only phyla with abundances more than 1% were counted for each city. **c** The community diversity reflected by Shannon index of three cities. Y-axis stands for the Shannon index calculated by the phyla distribution of a sample

We compared the community composition structure among three cities at phylum level predicted by MetaBinG2. Average proportions of phyla in each city were shown in Fig. 5a, and for each phylum (> 1% abundance), the overall percentage of samples containing it was shown in Fig. 5b. Combination of these two aspects showed the importance of one phylum. For example, if a phylum’s average proportion among samples was high and it also presented in most of samples, it meant that this phylum is predominant. The community diversity of each sample represented by Shannon Index was shown in Fig. 5c. The top 6 phyla of the average proportion in Sacramento were Streptophyta (~ 30%), Actinobacteria (~ 20%), Chordata (~ 10%), Ascomycota (~ 10%), Apicomplexa (~ 10%) and Bacillariophyta (~ 10%) (Fig. 5a). The average proportion of Streptophyta in Sacramento was higher than the other two cities (Fig. 5a). Over 80% samples in Sacramento contained these top 6 phyla (Fig. 5b). We also found that there was no significant difference among samples in Sacramento on phylum composition by Kruskal-Wallis test. Average proportion of each phylum from the 117 amplicon sequencing samples were shown in Additional file 1: Figure S5. Chordata and Actinobacteria in Boston samples (WGS) took the major proportions (Fig. 5a). Proteobacteria and Actinobacteria in Boston samples (amplicon) took the major proportions (Additional file 1: Figure S5). Actinobacteria was predominant in all these 141 samples of Boston city. In the same way, we found Proteobacteria was the predominant phylum in New York city’s samples (Fig. 5a-b). The phyla’s composition of samples among three cities was very different (Fig. 5a-b). Besides various predominant phyla of three cities, we calculated the Shannon Index for each sample and compare the difference of community diversity among three cities by Kruskal-Wallis test and Pairwise test with Bonferroni method. We found that the community diversity of New York was significantly different from the other two cities (*p*-value< 0.0001).

**Fig. 6:**
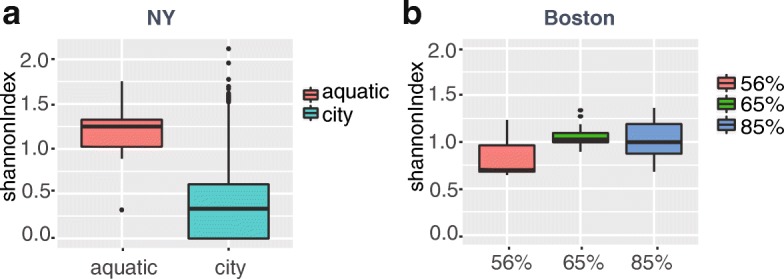
Relationship between factors and community diversity in NY and Boston sample. **a** Community diversities in NY samples are significantly related to the sampling location (*p*-value< 0.0001). **b** Community diversities in Boston samples are significantly related to humidity (*p*-value< 0.01). Y-axis stands for the Shannon index calculated by the phyla distribution of a sample

Furthermore, we found the community diversity were significantly different between samples labeled with ‘aquatic’ and ‘city’ in New York city (*p*-value< 0.0001) (Fig. 6). The former samples were sampled from canal, and the latter samples were sampled from parks or subway stations. The community diversity of samples between subway stations and parks have not significant difference. A significant relationship between community diversity and humidity was found in Boston samples (amplicon) (*p*-value< 0.01 with Kruskal-Wallis test). Especially for samples under 56% humidity, both material type and surface type were found to be related to the community diversity (Additional file 1: Figure S7). Community diversity of samples from seat or seat back with material of polyester is significantly higher than from other places with other material (*p*-value< 0.0001 Pairwise test) (Additional file 1: Figure S7).

At last, the relationship between the proportion of each phylum in one sample and the environmental factors for each city is estimated by p-value with Kruskal-Wallis test (Additional file 1: Figure S8).

## Discussion

MetaBinG2 has some advantages to classify a metagenome sequence dataset when we have little knowledge about it. The classification accuracy of current tools will be improved as more reference genomes are sequenced. There are less than 2 thousand microorganisms’ genomes were available on NCBI in 2012 [7], but today the number of available microorganism genomes is more than 7 thousand. However, the known microorganisms will be only a tiny fraction of all microorganisms in many environments for a long time. What’s more, the growing number of known genomes require more memory resource. The memory requirement of MetaBinG2 is much lower than other methods. Therefore, MetaBinG2 has a great potential to include many more genomes in the reference database than the other programs.

To accelerate the computing speed, most methods have been designed with parallelization. Both CLARK and metaCV provide the multi-thread mode. The popularity of multi-core CPU makes it possible to design CPU parallelized program. MetaBinG obtains the 1500-fold speed up compared to Phymm by using GPUs. MetaBinG2 added CPU parallelization in addition to the GPU parallelization, which made MetaBinG2 faster than MetaBinG.

For the MetaSUB data, MetaBinG2 was able to classify all samples in a few days. The results were compared to the previous study. For 24 Boston samples (WGS), Hsu et al. used KneadDATA v0.3 pipeline to remove low-quality sequences and human host sequences [22]. The average sequence depth decreased from 16.7 × 10^6^ to 9.8 × 10^6^ sequences per sample. In samples after pretreatment, Actinobacteria took the major proportion. In our analysis, the result was similar: ~ 30% of sequences were identified as Chordata and the top 2 phyla in terms of frequency were Chordata and Actinobacteria (both with abundance over 30%) (Fig. 5a). The previous study [22] suggested that microbial communities on transit surfaces were corresponding to local interactions with the human body and environmental exposures. In our results, we found the community diversity on seat and seat back was significantly higher than the other places (grips and touchscreens) when humidity is 56% (*p*-value< 0.0001 Pairwise test), and seat is relatively higher than seat back (Additional file 1: Figure S7). For New York samples, our results showed the similar community composition on phylum level with a previous study by Afshinnekoo et al. [23] (Additional file 1: Figure S6). For New York samples and Boston samples, we found humidity as a factor associated with the community diversity (Fig. 6). The places with higher humidity may have higher community diversity. For the Sacramento samples, it was reasonable that Streptophyta, Actinobacteria, and Chordata took the major proportions. These samples were from light rail stations, and the sampling locations were on the ground, where the surrounding vegetation is abundant. Although there is not significant difference among samples, some phyla’s changes among samples may give some useful information. For example, human traffic may be estimated with the result of MetaBinG2 classification (Additional file 1: Figure S9). Ticket machine in Archives Plaza (west) station and platform railing in station 6 have more Chordata sequences. The human traffic in these two stations may be higher than the other stations. In Archives Plaza (west) station, ticket machine has much more sequences from Chordata compared with bench and platform railing. It will be interesting to analyze the relationship of factors like the waiting time and the abundance of sequencing sequences in this station. In 8th & Capitol station, platform railing has more Chordata sequences, it may be caused by the surrounding facilities. For example, we found that the platform rail in this station is very close to a traffic light. The Chordata might be left by people waiting for traffic light. All this speculation should be verified in further analysis, but it implied a reasonable way to research and show the potential applications of MetaBinG2.

In addition to analyzing unknown environmental samples, like soil, water etc., MetaBinG2 can also be applied to compare two experiments or identify the changes between two experiments. For example, it can help identifying factors impacting the repeatability of an experiment or finding the source of contamination in a laboratory.

Sequence classification methods compared in this paper try to predict the source of each sequence, and these classification results can be subsequently used to analyze community composition structure. For community composition structure comparison, other than using sequence classification strategies, there are reference-free methods directly focused on differences among samples [3] and marker-based methods like MetaPhlAn2 [24] focused on community structure reconstruction rather than each sequence classification. Researchers should choose appropriate methods according to their own research goals.

## Conclusions

MetaBinG2 provides an effective way for us to understand the outline of the community composition structure of samples with little knowledge, and it has the potential to be applied to large-scale projects. With MetaBinG2, we could obtain the community composition structure of each sample in MetaSUB dataset within 3 days. The dominant phyla and community complexity are different among different cities. The community composition structure is significantly related with environmental factors like humidity.

## Reviewers’ comments

### Reviewer’s report 1: Eran Elhaik, Ph.D.,University of Sheffield, UK.

**Reviewer comments:** R1_S1, “In this manuscript, Qiao et al. present MetaBinG2, an upgraded method to MetaBinG, a method they published in an earlier paper. The new method, under certain conditions, is purported to be faster and more accurate than competing methods. The authors compare the new methods with established methods using two datasets. After establishing that the method is indeed an improvement, they apply it to two additional datasets (MetaSUB and Rumen microbiome).”

Author’s response: *Thanks.*

**Reviewer comments:** R1_1, “I appreciate the authors’ approach in first comparing their methods with competing methods and then applying it to two new datasets. I agree that it is conceivable that the new method is indeed an improvement and can help progress knowledge in the field.”

Author’s response: *Thanks.*

**Reviewer comments:** R1_2, “However, the paper is very poorly written and is unpublishable. I understand that English is not the authors’ first language and request them to make the necessary efforts to improve the quality of the work. The problems were not only with the language but also with the structure of the paper. I cannot possibly comment on all the writing problems with the manuscript.”

Author’s response: *Thanks for reviewer’s points about writing. We have revised the manuscript thoroughly and rearrange the article structure.*

**Reviewer comments:** R1_2, “In many places, I had difficulties understanding what the authors want to say.

1. The introduction is too long and read like results. It should be half its current size and written like proper introduction.

Author’s response: *Thanks for pointing this out. We have rewritten the introduction part (Background) it is more concise and better organized.*

**Reviewer comments:** R1_2 (2), “I do not understand the term mock dataset. Is it not unreal?”

Author’s response: *Mock dataset is between simulated dataset and real sequenced dataset. In simulated datasets, the source of each sequence is known. But in real dataset, it is not. A mock dataset was generated by sequencing (real sequencing, not simulation) of DNA extracted from a mixture of microbes with a predefined proportion. Although the exact source of each sequence is unknown, the candidate sources are known and, the approximate proportion of each microbe is also known. The mock dataset we used here was downloaded from HMP Mock Community. We have rewritten the introduction about mock dataset and one sentence has been added to introduce the mock data briefly as follows.*

“Another way to evaluate metagenomics analysis methods is using a mock dataset, which is generated by sequencing a mock community (a mixture of microbes with predefined proportions). In terms of similarity to the real-world data, a mock data is between simulation data and real-world metagenome sequencing data.”

**Reviewer comments:** R1_2 (3), “For each query sequence, a genome in the reference database with the minimum score is selected as its source genome” what score? You never mentioned any score. How is it being calculated?”

Author’s response: *Sorry for the misunderstanding. To avoid this misunderstanding, we have revised the manuscript to use “similarity score” instead of “distance”, “similarity”, or “score”. The similarity score represents the similarity between a query sequence and a genome in the reference database. It can be calculated by formula* (2)*.*

**Reviewer comments:** R1_2 (4), “I don’t understand how genomes with unknown organisms are being evaluated. It seems reasonable to me that the sample should go to its nearest relative.”

Author’s response: *Sequences from unknown organisms are predicted to their nearest relative genomes based on the similarity scores.*

**Reviewer comments:** R1_2 (5), “In summary, MetaBinG2 is helpful for researchers to learn about the overall community composition structure roughly in a short time when we have little knowledge about the environment.” “What does MetaBinG2 do when there is little information? Is it valuable? It will no doubt give the wrong results. Do we really need that? I am asking myself these questions to decide whether this manuscript is publishable. The authors should address these questions in the manuscript.”

Author’s response: *Thanks for the suggestion. We have revised the introduction and discussion session accordingly to address these issues. In general, it will be a long time that most sequencing reads are from unknown organisms for most environmental samples. However, a rough understanding about these samples is the first step to start before we get to know more.*

**Reviewer comments:** R1_3, “The authors should compare their MetaSUB results with those in the published papers.”

Author’s response: *Thanks for the suggestion. We added comparison of the MetaSUB results with previous published papers by Hsu* et al. [22] *and Afshinnekoo* et al. [23] *in the discussion part.*

**Reviewer comments:** R1_4, “Explain what GPUs are whenever you use them.”

Author’s response: *Thanks for pointing it out. We have added a brief introduction about GPUs in Background part.*

**Reviewer comments:** R1_5, “A million 100bp Illumina sequences can be classified in about 1 min with one GPU card. “ From this sentence it is unclear if you developed a tool for a computer or a sequencer.”

Author’s response: *Thanks for pointing this out. We have revised the sentence as “A million 100bp Illumina sequences can be classified in about 1 min on a computer with one GPU card.”*

**Reviewer comments:** R1_6, “K should be in italic”.

Author’s response: *Done.*

**Reviewer comments:** R1_7, “The authors list the known tool and explain about them, but in a different order than the one they used to present them. Why?”

Author’s response: *Thanks for pointing this out. Authors have rearranged the order and the orders are now consistent.*

**Reviewer comments:** R1_8, “BLAST should always be capitalized.”

Author’s response: *Done.*

**Reviewer comments:** R1_9, ““Moreover, most alignment-based methods, especially the blast-based methods are very slow. On the other hand, composition-based methods do not have such a high dependence on the known genomes, and most of them are fast” provide some numbers. slow and fast are relative terms.”

Author’s response: *Thanks for pointing it out. We have rewritten the Background and modify the vague statement as follows.*

“Kmer-alignment-based methods, like KRAKEN [9] and CLARK [10], have advantages both on speed and precision by using of exact-match database queries of kmers, rather than inexact alignment of sequences. For example, KRAKEN is about 900 times faster than Megablast (BLAST-based system) [9].”

“By contrast, composition-based methods, such as Phymm [11], NBC [12] and metaCV [13] depend less on reference genomes.”

“In summary, compared with alignment-based methods, composition-based methods have low dependence on the reference genomes, but at the same time, they are of low accuracy in general.”

**Reviewer comments:** R1_10, “You use microorganism, organism, and sometimes species interchangeably. They have different meaning.”

Author’s response: *We have revised the manuscript to make sure they were used in the right context with proper meaning.*

**Reviewer comments:** R1_11, “In the methods you first talk about the 2 reference datasets and then continue to give a lot of numerical details, which can be easily be presented in a table and the whole explanation about these datasets can be merged.”

Author’s response: *Thanks for pointed it out. We have merged the numerical details in the explanation about the two reference datasets and used* Table 1 *to show them.*

**Reviewer comments:** R1_12, ““In this mock dataset, some species are known dominant” what does it mean?”

**Fig. 7:**
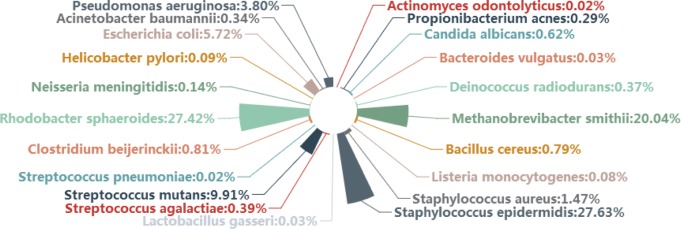
Community composition structure of mock dataset

Author’s response: *Sorry for the misunderstanding. This sentence should be “In this mock dataset, some species are dominant”. In the mock dataset we used in method evaluation, some microbes are obviously more frequent than others,* i.e.*, dominant. We draw a diagram to show the community composition structure of this dataset and rewrote the explanation about mock dataset to make the description clearer as follows* (Fig. 7).

“Another way to evaluate metagenomics analysis methods is using a mock dataset, which is generated by sequencing a mock community (a mixture of microbes with predefined proportions). In terms of similarity to the real-world data, a mock data is between simulation data and real-world metagenome sequencing data. We downloaded a mock dataset from HMP Microbiome Mock Community (HMMC, SRA run id: SRR072232). In this mock dataset, not all species are with the same proportions. Some species are dominant in this mock dataset (see details in Additional file 1: Table S1).”

**Reviewer comments:** R1_13, ““metagenomes with unknown organisms were simulated. For each simulated query datasets, several reference databases were created with all genomes at a specific taxonomy level a. excluded. “So, you didn’t simulate metagenome with unknown organisms, you used the simulated datasets … very confusing. Make it clearer and show a diagram.”

Author’s response: *We used clade exclusion experiment to mimic the scenario of unknown organisms in environmental sample. We draw a schematic diagram to illustrate the clade exclusion experiment as* Fig. 1*. It explains the clade exclusion experiment at order level.*

**Reviewer comments:** R1_14, ““which is achieved by cublas library on GPU.” Give reference. Couple of paragraphs below it is written CUBLAS. Pick one and stick with it.”

Author’s response: *Thanks for pointing this out. A reference has been added and CUBLAS is used for all places.”*

**Reviewer comments:** R1_15, “Wrong color in 3c and 3d (blue? Purple?)”

Author’s response: *Thank for pointing this out. The same color scheme has been applied to all four subfigures in* Fig. 4 *(the original Fig. 3) now.*

**Reviewer comments:** R1_16, ““Opportunistic pathogens are widely distributed in the samples“ what do you mean by “samples“? Do you mean between microorganism samples? you were just talking about cats, so this is confusing.”

Author’s response: *Thanks for pointing this out. Consider all reviews’ comments, the results about pathogens have been removed to avoid over interpolating of the sequencing data.*

### Reviewer’s report 2: Nicolas Rascovan, Ph.D., Mediterranee Infection Institute – Aix Marseille University, Marseille, France.

**Reviewer comments:** R2_S1, “Overall, I think that MetaBinG2 is a valuable method for the metagenomic field, since it is fast, it has very low memory use and seems to perform quite well on taxonomic classifications. The method is novel in the methodological approach that it uses (not dependent on alignments, uses HMM, the scoring is based on sample composition, it has low memory use, etc.) and I think that it will be well welcomed by the scientific community.

Author’s response: *Thanks.*

**Reviewer comments:** R2_S2, My biggest concern is the overall presentation of the manuscript, which has major stylistic flaws, lack of clarity and insufficient development in certain parts. Regarding the analyses, I think that the most widely used methods in the field (Kraken and Diamond-MEGAN) should be also compared with MetaBinG2 and that the comparative analyses of real metagenomic data (the rumen cow) should be improved. I found the results and conclusions from MetaSUB data a bit superficial. The discussion should be largely improved.”

Author’s response: *We have extensively revised the manuscript according to reviewers’ comments. DIAMOND has been added into method comparison and the result interpolation for MetaSUB has been improved. See more detailed information below.*

**Reviewer comments:** R2_1, “The manuscript by Qiao et al. presents a fast composition-based method to classify metagenomic reads taxonomically at different taxonomic levels by using Markov models to estimate the distance of a read to the organisms in a training set/database. The algorithm calculates a score of a read to all genomes in the database (assigning higher weights to the genomes in higher proportion in the sample) and finally assigns the taxonomic classification based on the genome with minimum score. The principal advantage highlighted by the authors is that the algorithm performs well in cases where the species (or higher taxa rank) of origin of a certain read is not present in the database (i.e., the method performs well “unknown organisms“). In addition, the MetaBinG2 has a much lower memory use than other methods. Although there are several tools already available for taxonomic classification of metagenomics reads, this is still a great and unsolved problem in metagenomics, and new tools using different approaches are always welcomed.”

Author’s response: *Thanks.*

**Reviewer comments:** R2_2, “Important note: It would have been much easier to make the revision if line numbers were correctly placed.”

Author’s response: *Thanks for pointing this out. Line numbers are correctly placed now.*

**Reviewer comments:** R2_3, “The English writing should be improved (e.g., weird grammar and wording). I would suggest to make the manuscript corrected by a native or fluid English spoken person before publication. For instance, I had hard times understanding many fragments of the text, just because of the way they were explained.”

Author’s response: *Thanks for points about writing. We have revised the manuscript extensively and rearrange the article structure as well.*

**Reviewer comments:** R2_4, “Page 2: “Moreover, most alignment-based methods, especially the blast-based methods are very slow“. Well, Kraken and Clark are not.”

Author’s response: *Thanks for pointing this out. We have rewritten the Background part and made the new description about categorization of sequence classification methods. The alignment-based methods were further divided into (i) Seed-and-extend algorithm-based methods like BLAST* [6] *and DIAMOND; (ii) Mapping-based methods, like MiCop; and (iii) Kmer-alignment-based methods, like Kraken and CLARK. Seed-and-extension alignment-based methods were slow in general while, mapping-based methods and Kmer-alignment-based methods were much faster. The description has been revised accordingly.*

**Reviewer comments:** R2_5, “Page 3, Lines 5-8: I think that it would be good to give a better explanation of the hypothesis underlying the MetaBinG2 method. Specifically, how does the method know a priori, which are the most abundant organisms in the samples when assigning weights?”

Author’s response: *Thanks for pointing it out. The detailed description about the hypothesis underlying MetaBinG2 is rewritten in Methods part as follows.*

“We designed MetaBinG2 based on an assumption that a query sequence is more likely from the organism with a larger proportion when the similarity scores of a query sequence to several organisms are similar.”

“The improvement of algorithm is reflected with the value of ω_*i*_. Each genome will be assigned with an initial value (an evenly distributed probability) at the beginning. These values will be updated when a classification is completed and as the prior knowledge added in the next classification. We obtained the final classification result when this genome weights vector ω is leveled off.”

**Reviewer comments:** R2_6, “In the formula for building the database, the F is not defined.”

Author’s response: *Sorry for the misunderstanding. We have added the definition for Fs. The corresponding part was rewritten as follows.*

“The transition probabilities from a state *m* to a state *n* of the genome *i* is calculated as following:

4 $$ {KMM}_{i, mn}={P}_i\left(\operatorname{}{O}_n|{O}_m\right)=\frac{F_i\left(\operatorname{}{O}_n|{O}_m\right)}{F_i\left({O}_m\right)} $$

Where *O*_*m*_and *O*_*n*_are oligonucleotides of length *k* with *k* − 1 bases overlapped, *F*_*i*_(*O*_*m*_) stands for the number of state *m* in genome *i*, *F*_*i*_(*O*_*n*_) stands for the number of state *n*.

in genome *i*, *F*_*i*_(*O*_*m*_| *O*_*n*_) stands for the number of state *m* followed by state *n* in genome *i*, and *P*_*i*_(*O*_*m*_| *O*_*n*_) represents the transition probability from the *O*_*m*_ to the *O*_*n*_ of the genome *i*.”

**Reviewer comments:** R2_7, “Methods: “The similarity was computed as the correlation between two composition structures“. Which statistical method was used for this and how was this calculated? “The consistency will be computed at each taxonomy level“: Was computed? All statistical methods used and in which cases were applied should be mentioned in the methods section.”

Author’s response: *Thanks for pointing it out. The description about comparison method has been added in manuscript as follows.*

“We used simulated dataset and mock dataset with reference dataset A to compare community composition structure prediction accuracy for several metagenome sequence classification tools. The consistency between a predicted community composition structure and the expected community composition structure was measured by cosine distances at different taxonomy levels.”

**Reviewer comments:** R2_8, “The k-size used in MetaBinG2 and the reason for choosing this size should be mentioned from the very beginning in the methods section, not just at the last sentence of the page 9, when the manuscript is almost over.”

Author’s response: *Thanks for pointing it out. We have rearranged the narrative order about this problem and explain k-size setting in ‘Method of MetaBinG2’.*

**Reviewer comments:** R2_9, “Legends for Figs. 2 and 3 should include the information of which methods are composition-based and alignment-based.”

Author’s response: *Thanks for pointing it out. We have added this information in the legends of Figs.* 3 and 4 *(the original Figs. 2 and 3).*

**Reviewer comments:** R2_10, “Since little matters the strategy used in taxonomic classification of metagenomics reads (i.e., composition-based or alignment-based) as long as the method works, I think that the comparison of MetaBinG2 to other methods, should also include some of the most widely used alignment-based methods, such as Kraken and Diamond-MEGAN. Even if MetaBinG2 does not outperform these methods, it would be interesting to understand the reasons for this and which are the limiting steps, so further methods can use this information to build upon these findings.”

Author’s response: *Thanks for the suggestions for method comparison. We have added DIAMOND in the comparison (Figs. **3 **and* *4*, *and Additional file **1**: Figure S2-4). However, KRAKEN was left out due to two reasons: 1) it uses similar strategy as CLARK, which performs better or at least comparable to KRAKEN; and 2) KRAKEN needs very large size of memory. Ounit* et al. *pointed out that when CLARK requires 40-42 GB memory, KRAKEN needs 120-140GB memory for classification. For our reference dataset A, CLARK requires more than 50 GB memory, which indicates that KRAKEN will need more than 140 GB.*

*Compared to MetaBinG2, DIAMOND showed better performance on over-prediction and comparable speed. Other conclusions remain unchanged.*

**Reviewer comments:** R2_10, “Page 7, Lines 7,8: From Fig. 2e, f, is clear that the method performs well at predicting the phylum level of reads when the genomes of the species, genus, family or order that are present in the query dataset were excluded from the reference database. I think that the sentence “In addition, the performance of … ” should be better explained, for instance by clearly stating that authors are particularly referring to phylum level classification. Also, I think that it would be nice if they could somehow show how is the performance at other taxonomic ranks, when different clade exclusion levels are used (e.g., how good is the classification at class or order level, when species or genus are excluded?). My point is that phylum level is not the only rank that matters, and it would be good to know at which levels (of clade exclusion AND taxonomic level classification) the performance of the method drops.”

Author’s response: *Thanks for the suggestion. We have added the evaluation of other taxonomy levels in Additional file **1**:* *Figure S2.*

**Reviewer comments:** R2_11, “Fig. 3a-b: I understand that community structures were estimated without clade exclusion. Do these correlations change in the different methods if clades are excluded? (e.g., excluding species and genera?)”

Author’s response: *Thanks for pointing it out. We added these results in Additional file **1**: Figure S3.*

**Reviewer comments:** R2_12, “Fig. 3 legend: “(d) The speed of four tools … “I don’t see this plot anywhere. There are two different references to (d) and it does not show any speed measurement. In think that authors forgot to add a plot here (and this is actually showed in Table 1).”

Author’s response: *Thanks for pointing it out. It was a mistake and we have corrected it. All speed data were shown in Table **2**.*

**Reviewer comments:** R2_13, “Fig. 3c-d and results about this (Page 7, Lines 14-18): It is not clear at all the explanation about what Fig. 3c-d is showing. The authors should better develop this. Moreover, they should also better explain what does the “over-prediction rates” metric shows. What I understand from the way is written now (“The over-prediction rate was computed as the percentage of predicted taxonomy items not included in the expected composition structure”) is that this metric somehow estimates miss-assignments, since it will calculate how many taxa were predicted that were not included in the initial dataset of origin. Looks like MetaBinG (first version) is pretty bad, with 75-80% of miss-assignments at species level, and MetaBinG2 between 0 and 25% between phylum and species (best performing method). All these results should be properly described in the text.”

Author’s response: *Thanks for the helpful advice. We have redefined the over-prediction rate as follows.*

“The over-prediction rate was computed as the percentage of predicted taxonomy items not included in the expected taxonomy items, i.e. the number of predicted taxonomy items not included in the expected composition structure divided by the total number of predicted taxonomy items.”

*We have also adjusted the figures to reflect how many taxa were predicted that were not included in the initial dataset of origin of each method*. *The original Fig. 3 has been moved to Fig.* *4*. *The Figure* *description has been revised as follows*.

“The over-prediction rates of the four tools with the simulated dataset and mock dataset were shown in Fig. 4c-d. The original genome of each read in the mock dataset is unknown, but the mock dataset has the known composition structure so that we can evaluate these tools’ performance on this dataset through over-prediction rates. The over-prediction rate is the ratio of predicted taxonomy items not included in the expected composition structure and all predicted results without ‘unclassified part’, which reflect how many taxa were predicted that were not included in the initial dataset of origin. MetaBinG (first version) is prone to predict more wrong taxa results with over 50% on genus level. The performance of MetaBinG2 is much better than MetaBinG (first version) and like DIAMOND with between 0 and 15% between phylum and genus.”

**Reviewer comments:** R2_14, “Results, “speed” section. I think that the fact that the memory use of MetaBinG2 is significantly lower than in Clark and MetaCV should be highlighted in the results section as well (not only in the discussion), since it represents a major advantage of the method.”

Author’s response: *Thanks for the suggestion. We have highlighted this by 1) adding Table* *2 **to show the memory requirements for all tools. We also described this in the results part as follows.*

“We applied these selected tools to a simulated dataset with 100 million reads of length 100 bp against the reference database A (2,606 genomes) on the same machine (see details in methods). The time was measured in two parts, the time for loading database and the time for classifying. Results showed that CLARK was the fastest, while MetaBinG2 was comparable with DIAMOND and slightly better than metaCV and MetaBinG in terms of speed (Table 2). The memory required for CLARK and metaCV was more than 50GB, for DIAMOND was 23GB but for MetaBinG and MetaBinG2 was less than 1GB. Therefore, MetaBinG2 has a great potential to include many more genomes in the reference database than the other programs.”

**Reviewer comments:** R2_15, “Additional file 1: Table S1: Only 4 orders were found in the whole rumen?”

Author’s response: *Thanks for pointing it out. This description was misleading. Hess* et al. [19], *generated 446 genome bins, and 15 of them were estimated to be near-complete draft genomes and were suggested to be successfully assembled. These 15 draft genomes were then assigned into four orders, Bacteroidales, Clostridiales, Myxococcales, and Spiochaetales.”*

**Reviewer comments:** R2_16, “Page 8: “We ran MetaBinG2, MetaCV and CLARK on this dataset”: the assemble data or the single reads?? Based on Additional file 1: Figure S2, it seems that the authors used single reads, but this information was then compared to the taxonomic composition inferred from the assemble data. I think that these two datasets are not really comparable, since the assembled data likely represents a small fraction of the real diversity in the sample. Additional file 1: Figure S2 actually shows how many more taxa are observed in the sample (by the three methods) compared to the assembled result. The limited taxonomic representation in the assembled data is not really representative of the metagenome diversity. I think I would chose a clearer example to show the performance of MetaBinG2 in “real datasets” (e.g., comparing to another single read analysis and/or more complex communities and using the same reference databases). I wonder which would be the classification of the contigs/scaffolds in the cited work from 2011, if they were reanalyzed with the much newer Reference databases A and B (maybe certain contigs that were initially unclassified and not mentioned in the work would be now classified). In fact, in this work from 2011, the authors simply used Blast against NCBI-nr to classify contigs. Wouldn’t it be better to instead of getting the taxonomic composition of the rumen sample from the information reported in the article, to just re-calculate the proportions using the same method (Blast) against the same databases (A and/or B)? Moreover, I would suggest that a more accurate way to do these analyses would be to get the contigs, taxonomically classify contigs de novo, map the reads on the contigs, estimate the abundance of the taxa in the contigs dataset (using contig coverage, for instance) and classify all the mapped reads with MetaBinG2 (and other methods) to see whether single read analyses correlates well with the information in the assembled data analysis. This way reads and contigs data can be directly compared.”

Author’s response: *The cow rumen dataset was described in methods part. The cow rumen dataset contains reads instead of contigs. The description of this dataset is as follows.*

“Cow rumen dataset.

We chose a real-world dataset which was generated from the cow rumen [19] (SRA runid: SRX034809). The sample was sequenced by Illumina GAIIx with sequence length of 125 bps. The total number of sequences is about 140 million.”

*Thanks for the suggestion about Additional file**1**: Figure S4 (Figure S2 in original order)*. *For read-world dataset*, *no methods can give the absolute correct classification, even using assemble method. The performance evaluation should be based on the simulated dataset or mock dataset. We use cow rumen dataset to show the result of each tool when they were applied on a real-world dataset with many unknown organisms. The original Fig. S2 was revised as Additional file **1**: Figure S4.*

**Reviewer comments:** R2_17, “Analyses of MetaSUB data: Chordata assigned reads most likely have a human origin. I would suggest to eliminate human reads from datasets before performing the taxonomic analyses. Like this, samples will be more comparable at the microbial composition level. If Figures a and b are both complementary and necessary, then the particular results that each of them indicate should be mentioned in the results section (a and b are referenced together, so it seems that they are both redundant for the information authors wants to highlight from their analyses).”

Author’s response: *Thanks for this suggestion. MetaBinG2 can classify not only microorganisms. The compositions of samples in MetaSUB are complicated. Therefore, we included some eukaryotic genomes such as animal and plant genomes in reference dataset B which was used for MetaSUB data analysis. MetaSUB dataset is used to test whether MetaBinG2 has the potential to be used for a large-scale project. So we did not eliminate human reads in our analysis. Subfigure a and b showed different aspect of the data. For the comparability, we divided Boston samples in two categories: 24 WGS sequencing samples and 117 amplicon sequencing samples. Only WGS sequencing samples are used to compare with other cities.*

*We have revised the description of Fig.* *5a-b* *to show the differences between a and b, as follows.*

“We compared the community composition structure among three cities at phylum level predicted by MetaBinG2. Average proportions of phyla in each city were shown in Fig. 5a, and for each phylum (> 1% abundance), the overall percentage of samples containing it was shown in Fig. 5b. Combination of these two aspects could show the importance of one phylum. For example, if a phylum’s average proportion among samples was high and it also presented in most of samples, it meant that this phylum is predominant.”

**Reviewer comments:** R2_18, “What is the difference between Fig. 5 and S4? Wouldn’t it be better to just present one of them?”

Author’s response: *Thanks for pointing it out. We have deleted Fig. S4 and adjusted Fig. 5 in* *Additional file **1* to *Figure S8*.

**Reviewer comments:** R2_19, “I don’t really see how the Fig. 5, Additional file 1: FigureS3 and S4 show that humidity and temperature were the main factors affecting community composition.”

Author’s response: *Thanks for pointing it out. In order to analysis the relationship between factors and community composition, we chose a more reasonable way as follows.*

“Furthermore, we found the community diversity were significantly different between samples labeled with ‘aquatic’ and ‘city’ in New York city (*p*-value< 0.0001) (Fig. 6). The former samples were sampled from canal, and the latter samples were from parks or subway stations. The community diversity of samples between subway stations and parks have not significant difference. A significant relationship between community diversity and humidity was found in Boston samples (amplicon) (*p*-value< 0.01 with Kruskal-Wallis test). Especially for samples under 56% humidity, both material type and surface type were found to be related to the community diversity (Additional file 1: Figure S7). Community diversity of samples from seat or seat back with material of polyester is significantly higher than from other places with other material (*p*-value< 0.0001 Pairwise test) (Additional file 1: Figure S7).”

**Reviewer comments:** R2_20, “I would suggest to eliminate all the discussion about pathogens in the samples. These results are not showed in the manuscript and since this is a very sensitive information, it would require an accurate and proper analysis and validation. Authors should just focus in discussing the contributions of the method and the results that are presented in the figures. The discussion section should be, therefore, largely improved.”

Author’s response: *Thanks for pointing it out. The discussion about the pathogens in the samples has been removed according to reviewers’ recommendations.*

**Reviewer comments:** R2_21, “Line 4, page 7: “While on other levels, its performance is not as good as CLARK and metaCV (Fig. 2a) “Is it? It does not seem to perform worse than metaCV in Fig. 2a. Y-axes in the Figs. 2 and 3 should have legends to understand what they show.”

Author’s response: *Thanks for pointing it out. We have rewrite this sentence and add the description about Y-axes in Figs*. *3**and* *4 **(Fig. 2-3 in original order) legends as follows.*

“MetaBinG2 had similar accuracy with CLARK and DIAMOND on phylum level, and showed obvious improvement compared to MetaBinG. While at species level, MetaBinG2 was not as good as CLARK and DIAMOND (Fig. 3a).”

“Fig.3

Accuracy evaluation with simulated dataset against reference databases with/without clade exclusion. Tested scenarios include: (a-b) no exclusion, (c-d) species level clade exclusion, and (e-f) all level of clade exclusion. In (a-b), all genomes in the query dataset were included in the reference database and query sequence length is 100 bp (a) and 250 bp (b); In (c-d) all species in the query dataset were excluded in the reference database. The accuracy was measured on clade levels from species to phylum (a-d). (e-f) accuracy evaluation at phylum level, and different levels of clades were excluded in the reference database. In (a, c, e) the sequence length is 100 bp, and in (b, d, f) the sequence length is 250 bp. Y-axis in all Fig.3 stands for the accuracy (see details in Methods). Here, CLARK and DIAMOND are alignment-based methods and the others are composition-based methods.”

“Fig. 4

Evaluation for community composition structure prediction ability of each tool. (a-b) The consistency between the predicted community composition structure and the true community composition structure. Y-axis stands for consistency level reflected by cosine value. In (a) the query dataset was simulated dataset, and in (b) the query dataset was mock dataset. (c-d) Over-prediction of each tools. The Y-axis stands for the proportion of query sequences assigned to genomes outside of the true source genomes (not include the unclassified part). In (c) the dataset was simulated dataset, and in (d) the dataset was mock dataset with known composition structure. Here, CLARK and DIAMOND are alignment-based methods and the others are composition-based methods.”

**Reviewer comments:** R2_22, “There are too many stylistic issues in the manuscript to be listed. Authors should consider getting assistance to write the final version of the manuscript.”

Author’s response: *Thanks for reviewer’s points. We have revised the manuscript extensively and, as a result, the quality of the manuscript has been improved significantly.*

### Reviewer’s report 3: Serghei Mangul, University of California, Los Angeles, USA

**Reviewer comments:** R3_1, “The paper is missing background about the importance of accounting for unknown organisms. How are the results from the unknown organism used in the analysis? Why people need to account for them, instead of just ignoring? This is not imminently clear from the text. How the results would be if we ignore the unknown organisms. The experiment where the MetaBin2 is run in mode ‘not accounting for unknown organism’ will be helpful. The authors are suggested to cite the paper discussing the unknown organisms: Mangul, Serghei, and David Koslicki. “Reference-free comparison of microbial communities via de Bruijn graphs.” Proceedings of the 7th ACM International Conference on Bioinformatics, Computational Biology, and Health Informatics. ACM, 2016.”

Author’s response: *Thanks for pointing it out. We have added the explanation about the importance of unknown organisms in Background. The reference-free method is a way to deal with samples with many unknown organisms, but the difference among samples don’t have taxonomy information. The unknown query sequences can be classified to their nearest relatives by MetaBinG2 instead of being ignored. The reference-free method has been mentioned in discussion part as follows.*

“Sequence classification methods compared in this paper try to predict the source of each sequence, and these classification results can be subsequently used to analyze community composition structure. For community composition structure comparison, other than using sequence classification strategies, there are reference-free methods directly focused on differences among samples [3] and marker-based methods like MetaPhlAn2 [24] focused on community structure reconstruction rather than each sequence classification. Researchers should choose appropriate methods according to their own research goals.

**Reviewer comments:** R3_2, “Line 10. I would suggest modifying the classification. K-mer based tools should not be classified alignment-based. As so, Kraken and CLARK should be classified as k-mer based and Megan as alignment-based.”

Author’s response: *Thanks for this suggestion. We have rewritten the description of current tools as follows.*

“Sequence classification is a crucial step in metagenome analysis. The methods for metagenome sequence classification can be divided into two categories: (1) alignment-based methods and (2) composition-based methods.”

“Alignment-based methods can be further divided into seed-and-extend alignment-based method, mapping-based methods and kmer-alignment based methods. Seed-and-extend alignment-based methods like BLAST [6] and DIAMOND [7], which classify a query sequence by finding the best alignment to a big database of reference genomes through sequence alignment methods.”

“Mapping-based methods are faster than seed-and-extend alignment-based methods because of the benefits from the mapping algorithm, while their sensitivity is very low in general, like MiCoP [8]. Kmer-alignment-based methods, like KRAKEN [9] and CLARK [10], have advantages both on speed and precision by using of exact-match database queries of kmers, rather than inexact alignment of sequences. For example, KRAKEN is 909 times faster than Megablast (BLAST-based system) [9].”

**Reviewer comments:** R3_3, “It is worth to mention marker-based tools like Metaphlan2 (MetaPhlAn2 for enhanced metagenomic taxonomic profiling. Duy Tin Truong, Eric A Franzosa, Timothy L Tickle, Matthias Scholz, George Weingart, Edoardo Pasolli, Adrian Tett, Curtis Huttenhower & Nicola Segata. Nature Methods 12, 902-903 (2015)) and another alignment tool MiCoP, which based on BWA alignment: LaPierre, Nathan, et al. “MiCoP: Microbial Community Profiling method for detecting viral and fungal organisms in metagenomic samples.” bioRxiv (2018): 243188. Besides the classes of microbiome analysis method, there is a class of reference-free method. One of them is: Mangul, Serghei, and David Koslicki. “Reference-free comparison of microbial communities via de Bruijn graphs.” Proceedings of the 7th ACM International Conference on Bioinformatics, Computational Biology, and Health Informatics. ACM, 2016.”

Author’s response: *Thanks for the suggestion. These methods have been descripted in discussion part as follows.*

“Sequence classification methods try to predict the source of each sequence, and these classification results can be subsequently used to analysis community composition structure, like MetaBinG2. Apart from sequence classification strategy, there are reference-free methods [3] and marker-based methods like MetaPhlAn2 [24] directly focus on difference among samples or community structure prediction rather than each sequence prediction. Researchers should choose appropriate method according to different goal.”

**Reviewer comments:** R3_4, “Please explain how composition-based are different from alignment based and k-mer based methods”

Author’s response: *Thanks for the suggestion. We rewrote the description of current tools.*

“Sequence classification is a crucial step in metagenome analysis. The methods for metagenome sequence classification can be divided into two categories: (1) alignment-based methods and (2) composition-based methods.”

“Alignment-based methods can be further divided into seed-and-extend alignment-based method, mapping-based methods and kmer-alignment based methods. Seed-and-extend alignment-based methods like BLAST [6] and DIAMOND [7], which classify a query sequence by finding the best alignment to a big database of reference genomes through sequence alignment methods.”

“Mapping-based methods are faster than seed-and-extend alignment-based methods because of the benefits from the mapping algorithm, while their sensitivity is very low in general, like MiCoP [8]. Kmer-alignment-based methods, like KRAKEN [9] and CLARK [10], have advantages both on speed and precision by using of exact-match database queries of kmers, rather than inexact alignment of sequences. For example, KRAKEN is 909 times faster than Megablast (BLAST-based system) [9].”

“However, for all these alignment-based methods, their accuracy drops dramatically when dealing with samples with many unknown organisms. By contrast, composition-based methods, such as Phymm [11], NBC [12] and metaCV [13] depend less on reference genomes.”

**Reviewer comments:** R3_5, “This statement needs further explanation. Line 30. “ benefiting from the conservative property of amino acid sequences “. Usually, matching nucleotides sequences are more conservative compared to matching aa sequences. since the reads are generated as nt sequences.”

Author’s response: *Thanks for the suggestion. We rewrote the description about metaCV as follows.*

“MetaCV uses k-mer frequency vectors of translated peptide sequences instead of the nucleotide sequences against the reference protein sequence database to determine the source organism.”

**Reviewer comments:** R3_6, “Line 38. Please provide number of samples for each city (*n*=?) and average number of reads per city and standard deviation”

Author’s response: *Thanks for this advice. All information was added in* Additional file 1: Table S2.

**Reviewer comments:** R3_7, “Definition of dataset A and B are confusing. Some intuition behind the choice of those datasets needs to be provided. If the purpose was to simulate the effect of species missing from the reference this needs to be clearly defined and explained. For example, what the % missing and was it only bacteria or other species as well?”

Author’s response: *The 2606 genomes in reference dataset A are all from microorganisms. Reference dataset B include more microorganisms genomes and even some eukaryotes. Reference dataset A is a subset of dataset B. Since some existing tools are memory demanding, dataset B was too big as reference database for some tools. We downloaded all bacterial reference genome sequences in an older and smaller dataset. We have revised the description of dataset A and B to address this issue.*

“**Reference dataset A.** Reference dataset A contains 2606 microbe genomes and the genome numbers at various taxonomy level are shown in Table 1. They were downloaded from NCBI website (ftp://ftp.ncbi.nlm.nih.gov/genomes/archive/old_refseq/Bacteria/ updated on June 2, 2015). Multiple databases were generated from this reference dataset A to evaluate CLARK, DIAMOND, metaCV, MetaBinG, and MetaBinG2. All reference databases in our analysis except for MetaSUB analysis were generated according to Reference dataset A.

**Reference dataset B.** Reference dataset B is a comprehensive reference dataset. It contains 7675 genomes, including 7459 from bacteria, 63 from eukaryotes, 153 from Archaea. These genomes were downloaded from NCBI genome database (ftp://ftp.ncbi.nlm.nih.gov/genomes/) on Mar 27, 2017.The bacterial genome numbers at various taxonomy levels are shown in Table 1. Reference dataset A is a subset of reference dataset B. A comprehensive database was generated from this reference dataset B for MetaBinG2 on the MetaSUB dataset.”

**Reviewer comments:** R3_8, “Accuracy definition is incorrect. According to https://en.wikipedia.org/wiki/Precision_and_recall, Accuracy includes FN which is not part of Sensitivity and Precision.”

Author’s response: *The definition of accuracy we adopted in this paper was not the same as the one shown in this link. Since a large portion of the sequences may be classified as unknown by existing tools, we adopted the accuracy definition presented in this paper to deal with the unknown organisms. We believe it is a fair and reasonable measurement for our comparison.*

**Reviewer comments:** R3_9, “Running time, CPU usage, and CPU time needs to be added.”

Author’s response: *Thanks for pointing it out. We have added this information as follows* (Table 2)*.*

**Reviewer comments:** R3_10, “p.9 line 4. Definition of K-L divergence needs to be explained. Ideally, it purposes and rationale of using this metric needs to be explained”

Author’s response: *Thanks for pointing this out. Shannon index of one city was used to represent the community diversity of a sample. Shannon index distributions in samples of cities were used to compare the difference of sample’s community diversity among cities instead of K-L divergence. Definitions as well as the rational of using them have been added in the methods. Shannon index distribution is clearer to show the difference among cities’ samples.*

**Reviewer comments:** R3_11, “p. 9. line 8. The paper claims the highest complexity of Sacramento samples. Was this measure normalize by the total number of reads. Ideally, one would subsample each sample to bring all sample from different sample to the same number of reads.”

Author’s response: *The community complexity was measured by Shannon index, which was calculated by the proportions instead of the raw frequencies. Therefore, all samples have been normalized before comparison.*

**Reviewer comments:** R3_12, “Results obtained based on NY and Boston sample needs to compared to the publication originally introducing those. Is the paper able to confirm the results of the original papers? How was mush novel found due to the novel method?”

Author’s response: *Thanks for the suggestion. Comparison with published results of MetaSUB has been added in discussion part. Details can be found as follows.*

“The results were compared to the previous study. For 24 Boston samples (WGS), Hsu et al. used KneadDATA v0.3 pipeline to remove low-quality reads and human host sequences [22]. The average sequence depth decreased from 16.7 × 106 to 9.8 × 106 reads per sample. In samples after pretreatment, Actinobacteria took the major proportion. In our analysis, the result was similar: ~ 30% of reads were identified as Chordata and the top 2 phyla in terms of frequency were Chordata and Actinobacteria (both with abundance over 30%) (Fig. 5a), The previous study [22] suggested that microbial communities on transit surfaces are corresponding to local interactions with the human body and environmental exposures. In our analysis result, we found the community diversity on seat and seat back was significantly higher than the other places (grips and touchscreens) when humidity is 56% (*p*-value< 0.0001 Pairwise test), and seat is relatively higher than seat back (Additional file 1: Figure S7). For New York samples, our results showed the similar community composition on phylum level with a previous study by Afshinnekoo et al. [23] (Additional file 1: Figure S6). For New York samples and Boston samples, we found humidity as a factor associated with the community diversity (Fig. 6). The places with higher humidity may have higher community diversity. For the Sacramento samples, it was reasonable that Streptophyta, Actinobacteria, and Chordata took the major proportions. These samples were from light rail stations, and the sampling locations were on the ground, where the surrounding vegetation is abundant. Although there is not significant difference among samples, some phyla’s changes among samples may give some useful information. For example, human traffic may be estimated with the result of MetaBinG2 classification (Additional file 1: Figure S9).”

**Reviewer comments:** R3_13, “Results about pathogens are important. How confident authors are that those results are not FP. Pathogens originally reported in NY study, are actually FP, as was suggested here: https://www.nature.com/articles/nbt.3868, Living in a microbial world. The question of how probable those are FP needs to be addressed”

Author’s response: *Thanks for this point. The results about pathogens have been removed in order to avoid over interpolating of the sequencing data.*

**Reviewers’ comments (for the revision) RR_1:** “The authors have well addressed most of my comments and I don’t have much else to say about the scientific aspects of the manuscript. The method looks good, they show a clear improvement at different levels compared to previous methods and the results presented reflect well its performance. However, the manuscript cannot be published in the current form. There are so many issues in the general presentation of the manuscript, that is really pointless to put them in a list. I strongly recommend the authors to get assistance or work much more intensively on this. I will just list a few comments in the “Minor Issues” text box, which I made while reading the manuscript. These are merely examples, but the authors should be aware that it is only very few from many flaws in the texting of the manuscript.

Author’s response: *Thanks for reviewer’s suggestion. We have revised the manuscript again.*

Minor issues

**Latest reviewer’s comments:** RR_2_1, “Line numbers were not added, as they say in the response to reviewers, which still makes commenting the manuscript very complicated.”

Author’s response: *Done.*

**Reviewer comments:** RR_2_2, “I find the first part of the introduction (about metagenomics) not really relevant for purpose of this work, or the applications of the method. I think that the first paragraph can be simply deleted, for clarity.”

Author’s response: *MetaBinG2 classifies all sequences of samples rather than a few of them and ignore unknown organisms. This part introduces the importance of unknown organisms in many researches. These researches required a tool like MetaBinG2 to give an outline of a sample. The introduction for the importance of unknown organisms was also suggested by Reviewer 3 to make the background more substantial.*

**Reviewer comments:** RR_2_3, “MEGAN is not a seed-and-extend classification method per se. Is a visualization software for classified sequences (either by Blast or DIAMOND). I think that in the description of other available methods, what they do and what their flaws are could be a bit improved, to be clearer and more accurate.”

Author’s response: *We modified the description about methods mentioned accordingly in the introduction part.*

**Reviewer comments:** RR_2_4, “In the exclusion method, for subsets of dataset A, it is not clear which species, genus, orders, etc. excluded in each case, from (2) to (6). How many of each? The Fig. 1 does not really help on this. How many genomes are “condensed” within each (+) sign?”

Author’s response: *We added the details of each excluded database in the text. More information about simulated dataset is descripted in* Additional file 1: Figure S1.

**Reviewer comments:** RR_2_5, “Is not necessary to copy and paste a definition of Shannon index, which is widely used in metagenomics. Just how do they use it in the manuscript (which is not explained in M&M).”

Author’s response: *Done.*

**Reviewer comments: RR_**2_6, “The sentence about CUBLAS is duplicated in two consecutive paragraphs. This was already mentioned by Reviewer 1 in the first revision.”

Author’s response: *Thanks, one has been removed.*

**Reviewer comments: RR_**2_7, “Figure legend 3: there are redundant phrases (same information mentioned twice).”

Author’s response: Fig. 3 *has six subfigures labeled with (a)-(f). We mentioned the same information twice to avoid ambiguity.*

**Reviewer comments: RR_**2_8, “Fig. 4a and b are not mentioned in the text Which were the results of the Kruskal-Wallis test (they only say that it was not significant).”

Author’s response: *The description for* Fig. 4a and b *has been revised in the results part. Kruskal-Wallis test was used to analyze MetaSUB data* (Figs. 5 and 6)*.*

## Additional files

Additional file 1:  Supplementary results. (DOCX 482 kb)

Additional file 2: Sequence classification results of MetaSUB data from Boston. (XLSX 71 kb)

Additional file 3: Sequence classification results of  MetaSUB data from New York. (XLSX 2515 kb)

Additional file 4: Sequence classification results of MetaSUB data from Sacramento. (XLSX 16 kb)

## Abbreviations

HMMC: HMP Microbiome Mock Community
MetaSUB: Metagenomics & Metadesign of Subways & Urban Biomes
NY: New York
