# Supplementary material for: MetaBinG2: a fast and accurate metagenomic sequence classification system for samples with many unknown organisms
Source: Biol Direct. 2018 Aug 22;13:15. doi: 10.1186/s13062-018-0220-y (PMC6104016; doi:10.1186/s13062-018-0220-y)
Supplement: Supplementary file 1 — Supplementary results. (DOCX 482 kb) [file 13062_2018_220_MOESM1_ESM.docx]

Supplementary Data for:

**MetaBinG2: a fast and accurate metagenomic sequence classification system for samples with many unknown organisms**

Yuyang Qiao^1^, Ben Jia^1,2^, Zhiqiang Hu^1^, Chen Sun^1,2^, Yijin Xiang^4^, Chaochun Wei^1,2,3,$^

1. Department of Bioinformatics and Biostatistics, School of Life Sciences and Biotechnology, Shanghai Jiao Tong University, Shanghai, China, 200240
2. Shanghai Center for Bioinformation Technology, Shanghai, China, 201203
3. Shanghai Center for Systems Biomedicine, Shanghai Jiao Tong University, Shanghai, China, 200240
4. School of Medicine, Shanghai Jiao Tong University, Shanghai, China, 200025


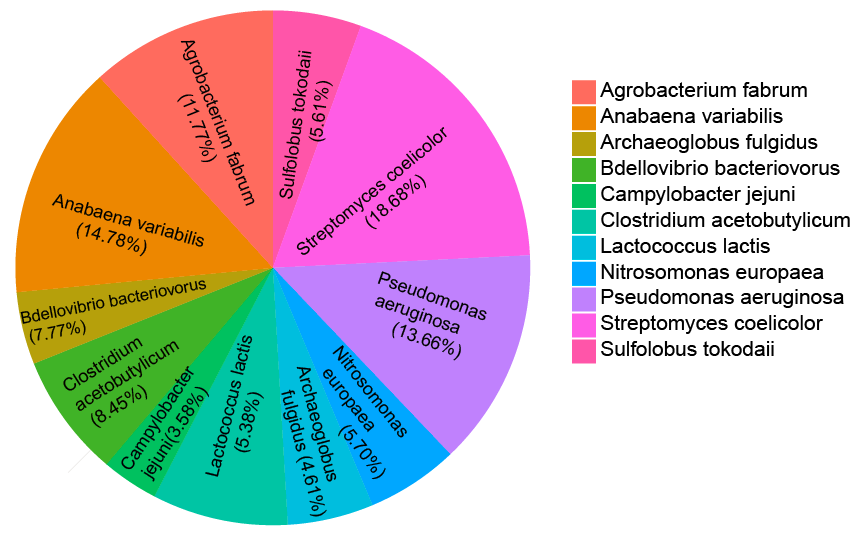


**Figure S1. The species abundance of** S**imulated data simulated by NeSSM.**

**Table S1. The species abundance of Mock data.**

| Organism and Repository number | gDNA mass | Estimated sequence percentage |
| --- | --- | --- |
| Acinetobacter baumannii ATCC 17978 | 1.60E-11 | 3.38E-03 |
| Actinomyces odontolyticus ATCC 17982 | 7.82E-13 | 1.65E-04 |
| Bacillus cereus ATCC 10987 | 3.73E-11 | 7.87E-03 |
| Bacteroides vulgatus ATCC 8482 | 1.52E-12 | 3.21E-04 |
| Candida albicans ATCC MY-2876 | 2.92E-11 | 6.16E-03 |
| Clostridium beijerinckii ATCC 51743 | 3.81E-11 | 8.04E-03 |
| Deinococcus radiodurans DSM 20539 | 1.76E-11 | 3.71E-03 |
| Enterococcus faecalis ATCC 47077 | 2.22E-13 | 4.68E-05 |
| Escherichia coli ATCC 700926 | 2.71E-10 | 5.72E-02 |
| Helicobacter pylori ATCC 700392 | 4.50E-12 | 9.49E-04 |
| Lactobacillus gasseri DSM 20243 | 1.53E-12 | 3.23E-04 |
| Listeria monocytogenes ATCC BAA-679 | 3.98E-12 | 8.40E-04 |
| Methanobrevibacter smithii ATCC 35061 | 9.50E-10 | 2.00E-01 |
| Neisseria meningitidis ATCC BAA-335 | 6.87E-12 | 1.45E-03 |
| Propionibacterium acnes DSM16379 | 1.39E-11 | 2.93E-03 |
| Pseudomonas aeruginosa ATCC 47085 | 1.80E-10 | 3.80E-02 |
| Rhodobacter sphaeroides ATCC 17023 | 1.30E-09 | 2.74E-01 |
| Staphylococcus aureus ATCC BAA-1718 | 6.97E-11 | 1.47E-02 |
| Staphylococcus epidermidis ATCC 12228 | 1.31E-09 | 2.76E-01 |
| Streptococcus agalactiae ATCC BAA-611 | 1.83E-11 | 3.86E-03 |
| Streptococcus mutans ATCC 700610 | 4.70E-10 | 9.92E-02 |
| Streptococcus pneumoniae ATCC BAA-334 | 8.11E-13 | 1.71E-04 |


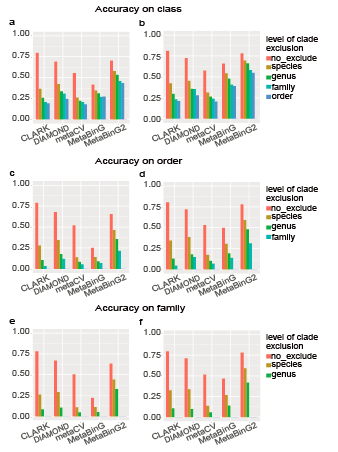


**Figure S2. Accuracy evaluation at various taxonomy levels when different levels of clades were excluded in the reference database.** Accuracy evaluation at (**a**-**b**) class level, (**c**-**d**) order level, and (**c**-**d**) family level. In (**a**, **c**, **e**) the sequence length is 100 bp, and in (**b**, **d**, **f**) the sequence length is 250 bp. Here, CLARK and DIAMOND are sequence alignment-based methods and the others are composition-based methods.


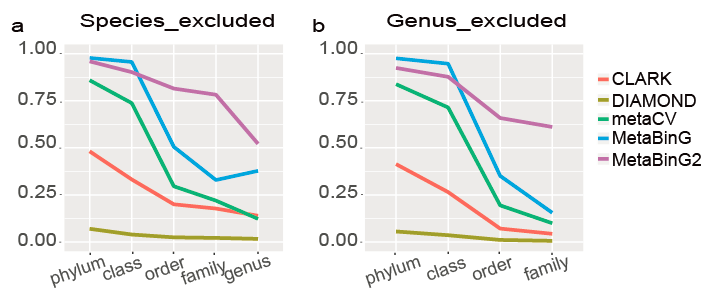


**Figure S3. Evaluation for community composition structure prediction ability of each tool on simulated dataset with incomplete databases.** The consistency between the predicted community composition structure and the true community composition structure. Y-axis stands for consistency level reflected by cosine value. In (**a**) the database is under species clade exclusion, and in (**b**) the database is under genus clade exclusion.


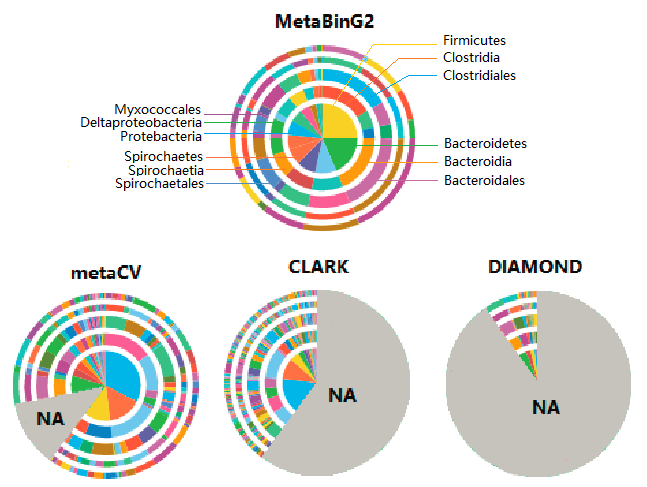


**Figure S4. Community composition structure results of cow rumen dataset with different software.** The results of CLARK, DIAMOND, metaCV and MetaBinG2 displayed by pie figure from phylum (inside) to genus (outside). The part of unclassified sequences represented by 'NA'.

**Table S2. Average number of sequences per city and standard deviation.**

| City | #Samples | Average of #Sequences | Standard deviation of #Sequences |
| --- | --- | --- | --- |
| Boston (WGS) | 24 | 18M | 15M |
| Boston (amplicon) | 117 | 0.05M | 0.04M |
| NY | 1451 | 3M | 4M |
| Sacramento | 18 | 44M | 2M |

In Table S2, ‘#Samples’ stands for the number of samples and ‘#Sequences’ stands for the number of sequences in one sample.


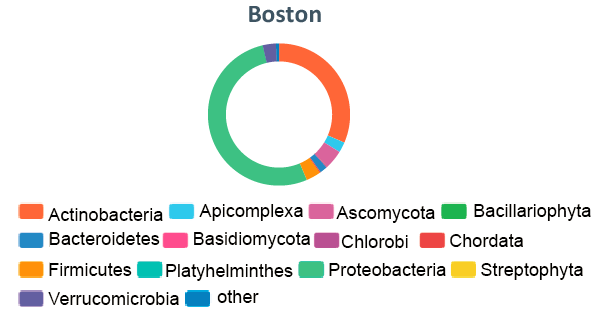


**Figure S5. Average community abundance of Boston city at phylum level.** There are 117 amplicon sequencing samples from Boston city used to calculate each phylum’s average community abundance.


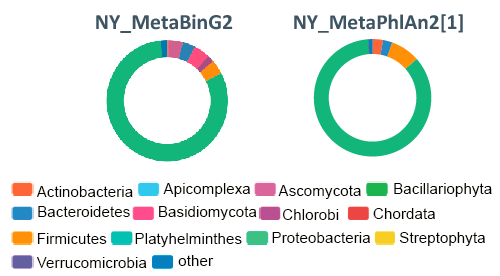


**Figure S6. The Comparison between MetaBinG2’s classification results and previous work on samples from New York city at phylum level.** The former ‘NY_MetaBinG2’ represent average community abundance of New York city at phylum level analysis with MetaBinG2. The second one ‘NY_MetaPhlAn2’ represent average community abundance of New York city at phylum level analysis with MetaPhlAn2 in previous work ^[1]^.


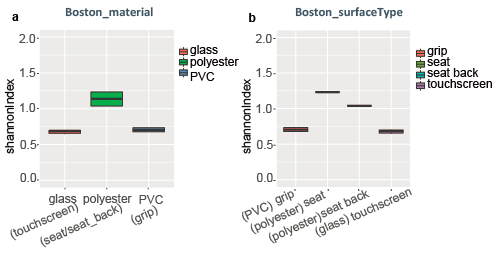


Figure S7. Relationship between factors and community complexity of Boston samples. In Boston samples (amplicon), community diversity of samples from seat or seat back with material of polyester is significantly higher than from other places with other material (p-value<0.0001 Pairwise test).


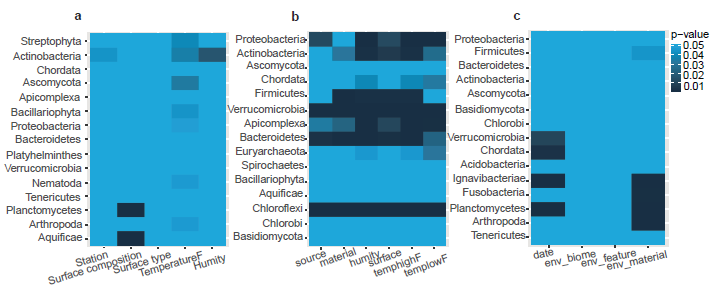


**Fig S8. Relationship between factors and community composition structures.** The heatmaps were used to show whether a factor has a significant influence on a phylum's abundance in three cities: (**a**) Sacramento, (**b**) Boston, and (**c**) NY. ­Only the top 15 most abundant phyla were displayed. The deeper color represents the lower p-value with Kruskal-Wallis test.

**
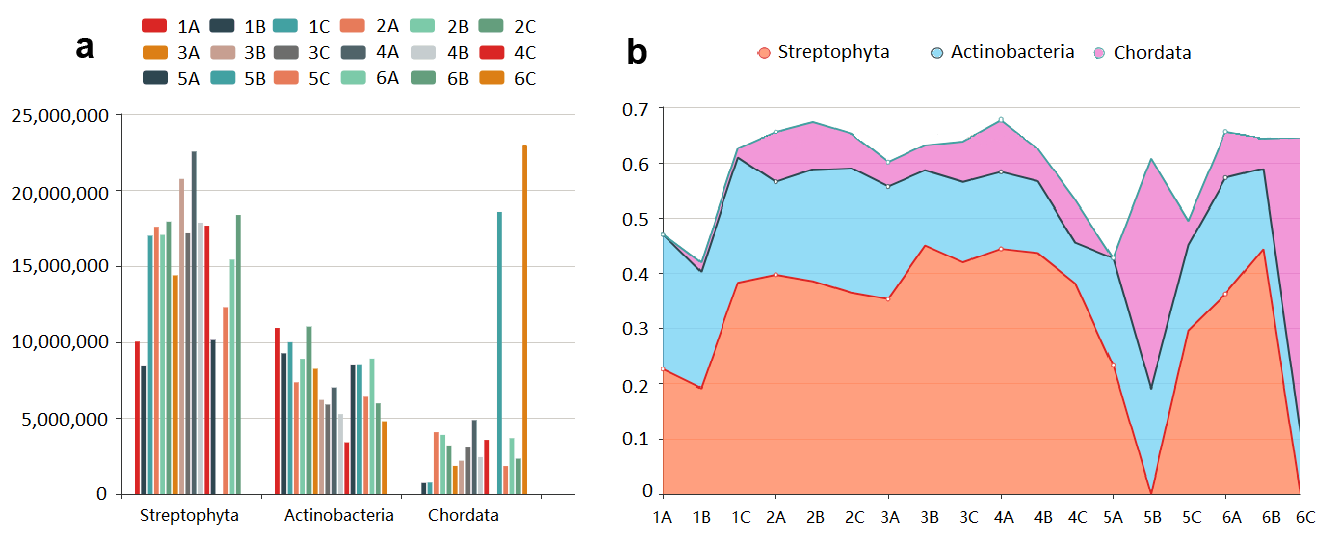
**

**Figure S9. The distribution of three predominant phyla in Sacramento city.** (**a**) The predicted sequences number of a predominant phylum in each sample. There are 18 samples labeled with (1A-6C) and assigned with different colors. (**b**) The proportion of three predominant phyla in 18 samples.

**Table S3. Description for Sacramento samples.**

| Sample | Station | Surface type |
| --- | --- | --- |
| 1A | St. Rose of Lima Park | Bench |
| 1B | St. Rose of Lima Park | Ticket Machine |
| 1C | St. Rose of Lima Park | Plateform railing |
| 2A | 9th & K | Bench |
| 2B | 9th & K | Ticket Machine |
| 2C | 9th & K | Plateform railing |
| 3A | 8th & O (west) | Bench |
| 3B | 8th & O (west) | Ticket Machine |
| 3C | 8th & O (west) | Plateform railing |
| 4A | 8th & O (east) | Bench |
| 4B | 8th & O (east) | Ticket Machine |
| 4C | 8th & O (east) | Plateform railing |
| 5A | Archives Plaza (west) | Bench |
| 5B | Archives Plaza (west) | Ticket Machine |
| 5C | Archives Plaza (west) | Plateform railing |
| 6A | 8th & Capitol | Bench |
| 6B | 8th & Capitol | Ticket Machine |
| 6C | 8th & Capitol | Plateform railing |

**Reference**

1. Afshinnekoo E, Meydan C, Chowdhury S, et al. *Geospatial Resolution of Human and Bacterial Diversity with City-Scale Metagenomics*. Cell Systems, 2015, 1(1):72.
